# First person – Scott Collum

**DOI:** 10.1242/dmm.040295

**Published:** 2019-05-15

## Abstract

First Person is a series of interviews with the first authors of a selection of papers published in Disease Models & Mechanisms (DMM), helping early-career researchers promote themselves alongside their papers. Scott Collum is first author on ‘Adenosine and hyaluronan promote lung fibrosis and pulmonary hypertension in combined pulmonary fibrosis and emphysema’, published in DMM. Scott is a PhD student in Harry Karmouty-Quintana's lab at the Department of Biochemistry and Molecular Biology, McGovern Medical School, University of Texas Health Science Center at Houston, USA, investigating the molecular mechanisms involved in the initiation and development of chronic lung diseases.


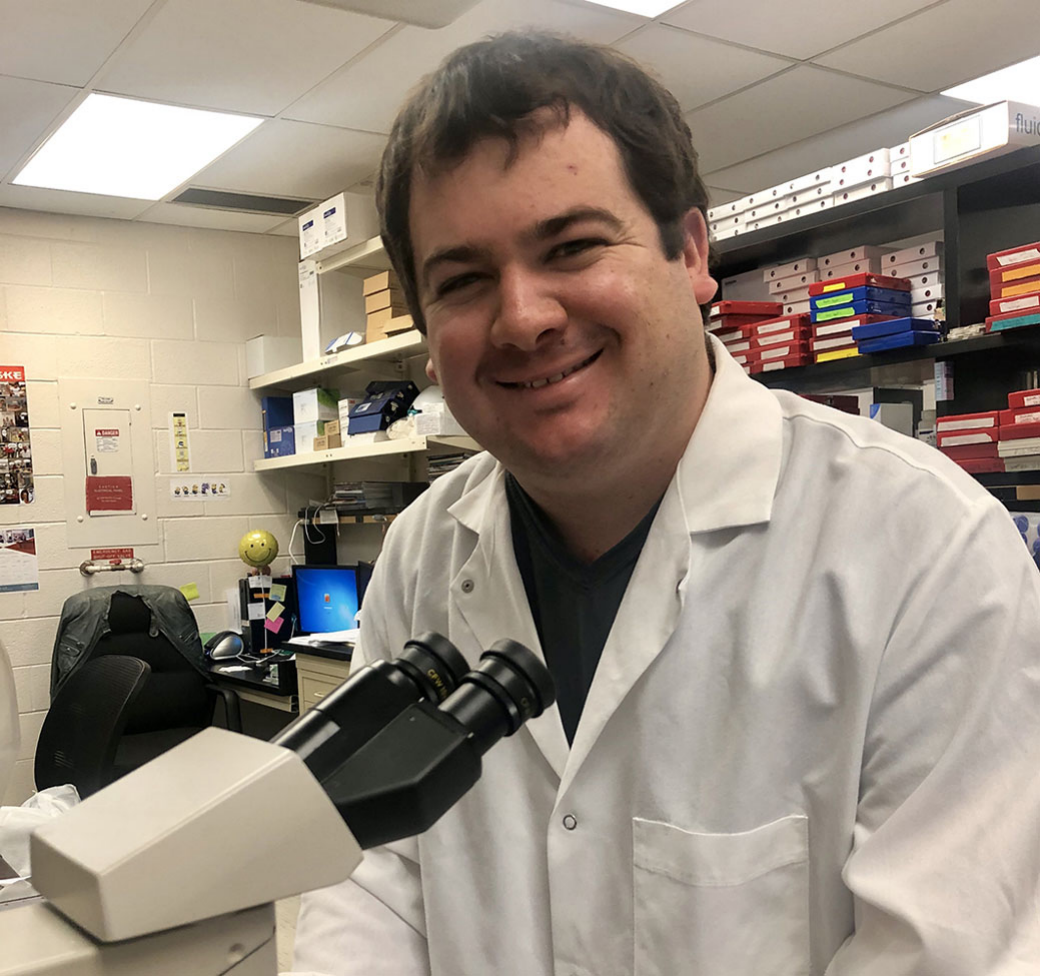


**Scott Collum**

**How would you explain the main findings of your paper to non-scientific family and friends?**

Combined pulmonary fibrosis and emphysema (CPFE) is a disease that involves both destruction of the lung tissue (emphysema) and inappropriate scarring of the lung tissue (fibrosis). These changes, along with an increase of the blood pressure in the lungs known as pulmonary hypertension, are a deadly combination with no drug treatment available. In tissues from patients with CPFE and pulmonary hypertension, we discovered that the adenosine receptor ADORA2B was elevated in sick lungs compared to healthy lungs. This increased signaling also led to an increase in HAS3 protein, which makes hyaluronan, a molecule that is part of the scaffolding of the lung. In our mouse model of CPFE and pulmonary hypertension, in which adenosine accumulates, we saw similar changes in HAS3 levels. Using 4-methylumbeliferone (4MU), which reduces hyaluronan, we improved the lungs in our mouse model. 4MU is a US Food and Drug Administration (FDA)-approved drug not previously used in the investigation of CPFE and pulmonary hypertension. This could lead to exciting possibilities for future studies and treatment.

**What are the potential implications of these results for your field of research?**

My research shows a novel pathway involved in this complicated and hard-to-model chronic lung disease. The use of this unique model of CPFE allowed us to determine the role of adenosine signaling in the development of disease. Of particular interest is the discovery of HAS3-expressing macrophages. Hyaluronan synthases have been shown to play a role in chronic lung diseases in previous work by our lab and others. Macrophages are also an important driver in many mechanisms of chronic lung diseases. It has not previously been shown that macrophages could play an important role in the accumulation of hyaluronan in the damaged areas of the lung. The finding that 4MU treatment will reduce this activity of macrophages and lead to reduced features of CPFE in our model allows for the continued investigation of hyaluronan as a driver of this disease and offers a potential treatment for patients with CPFE.

“The finding that 4MU treatment will reduce […] features of CPFE in our model […] offers a potential treatment for patients with CPFE.”

**What are the main advantages and drawbacks of the model system you have used as it relates to the disease you are investigating?**

The *Ada*^−/−^ mouse model recapitulates the features of CPFE with secondary pulmonary hypertension quite well, with readily apparent fibrotic deposition and airway enlargement by histology and increased pulmonary vascular pressures. Another advantage is that ADA is supplemented for these mice during development in the form of PEG-ADA. This allows for reduction of ADA at a controlled time and gradual accumulation of adenosine in a more chronic manner. Unfortunately, the supplement of PEG-ADA must be used in maintenance of the colony and for several months for each experimental mouse. In addition, important environmental stimuli, such as cigarette smoke or environmental particulate matter exposure, that are associated with CPFE do not form part of the pathophysiological response in our model.

**What has surprised you the most while conducting your research?**

I was most surprised by how well this new model recapitulated the key features of CPFE, both the obliteration of emphysema and the fibrotic deposition with secondary pulmonary hypertension. The model even recapitulates the novel findings of HAS3-positive macrophages.

**Figure:**
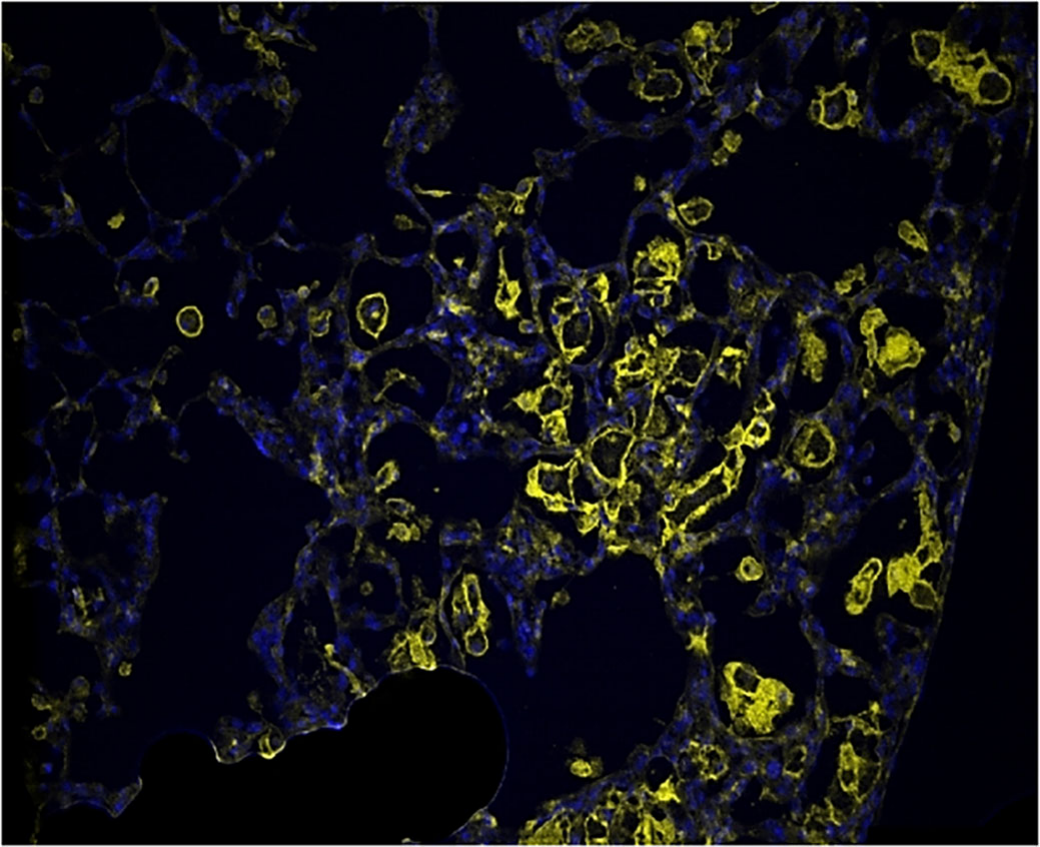
**Marked increase in the number of large macrophages after withdrawal of PEG-ADA in the mouse model of CPFE.** Yellow staining of F4/80 with DAPI.

**Describe what you think is the most significant challenge impacting your research at this time and how will this be addressed over the next 10 years?**

The greatest challenges in the chronic lung disease field are the relatively small number of animal models for each disease and the difficulty in obtaining patient tissues. As this paper and others in the field have shown, many people are working to develop new models of chronic lung disease that recapitulate more accurately the symptoms and mechanisms involved in the human disease progression. In addition, collaborative efforts have begun to allow for greater access to human samples, which greatly help the translation of exciting findings in the novel animal models.

**What changes do you think could improve the professional lives of early-career scientists?**

I believe that two separate initiatives would improve early-career scientist training. First, an increased focus on funding predoctoral fellowships would allow for an important first step in gaining independence in an academic setting and would propel this career path forward. Second, a widespread collaborative training program with industry would allow for a clear career path for those that transition out of academia. Together these would reduce the uncertainty that many early-career scientists feel.

“I believe that two separate initiatives would improve early-career scientist training. First, an increased focus on funding predoctoral fellowships […] Second, a widespread collaborative training program with industry.”

**What's next for you?**

I am finishing a research project on RNA processing in pulmonary hypertension that may help to explain how hyaluronan synthases are regulated in disease. I am preparing to defend my thesis on this project. I am also searching for postdoctoral positions with a continued focus on translational research.
